# Neuropathy and efficacy of once weekly subcutaneous bortezomib in multiple myeloma and light chain (AL) amyloidosis

**DOI:** 10.1371/journal.pone.0172996

**Published:** 2017-03-09

**Authors:** Surbhi Sidana, Mayur Narkhede, Paul Elson, Debbie Hastings, Beth Faiman, Jason Valent, Christy Samaras, Kimberly Hamilton, Hien K. Liu, Mitchell R. Smith, Frederic J. Reu

**Affiliations:** 1 Department of Internal Medicine, Cleveland Clinic, Cleveland, Ohio, United States of America; 2 Department of Quantitative Health Sciences, Cleveland Clinic, Cleveland, Ohio, United States of America; 3 Department of Cancer Center Research, Cleveland Clinic, Cleveland, Ohio, United States of America; 4 Department of Hematology & Oncology, Taussig Cancer Institute, Cleveland Clinic, Cleveland, Ohio, United States of America; Institut national de la recherche scientifique, CANADA

## Abstract

**Introduction:**

Randomized studies have shown that bortezomib (BTZ) can be given weekly via intravenous (IV) route or twice weekly via subcutaneous (SC) route with lower neuropathy risk and no loss of anti-myeloma efficacy compared to original standard IV twice weekly schedule. Weekly SC should therefore yield the best therapeutic index and is widely used but has not been compared to established administration schedules in the context of a clinical trial.

**Methods:**

Comprehensive electronic medical record review was done for disease control and neuropathy symptoms of 344 consecutive patients who received their first BTZ-containing regimen for myeloma or AL amyloidosis before or after we changed to SC weekly in December 2010. Univariate and multivariable analyses were carried out that adjusted for age, underlying disease, concurrently used anticancer agents, underlying conditions predisposing to neuropathy, and number of prior regimens compared SC weekly to other schedules.

**Results:**

Fifty-three patients received BTZ SC weekly, 17 SC twice weekly, 127 IV weekly and 147 IV twice weekly. Risk for neuropathy of any grade was higher with other schedules compared to SC weekly (44.3% vs. 26.9%, p = 0.001) while response rate was similar (72.1% vs. 76.6%, respectively, p = 0.15). Multivariable analyses upheld higher neuropathy risk (Odds ratio 2.45, 95% CI 1.26–4.76, p = 0.008) while the likelihood of not achieving a response (= partial response or better) was comparable (Odds ratio 1.25, 95% CI 0.58–2.71, p = 0.56) for other schedules compared to SC weekly, respectively. Lower neuropathy risk translated into longer treatment duration when BTZ was started SC weekly (p = 0.001).

**Conclusions:**

Weekly SC BTZ has activity comparable to other schedules and causes low rates of neuropathy.

## Introduction

Bortezomib (BTZ) with glucocorticoids or in combination with other chemotherapeutic agents is standard treatment for newly diagnosed, relapsed and refractory multiple myeloma and light chain (AL) amyloidosis.[1–7] A major adverse effect associated with BTZ treatment is dose-dependent peripheral neuropathy (PN)[6], mainly sensory. While PN improves in the majority of patients after treatment is stopped, this may take months or even years in some patients. Recovery may not be complete; leaving patients with chronic PN and neuropathic pain.[6,8–10] The pathogenesis of neuropathy is not entirely clear, although it has been proposed that BTZ causes direct dorsal root ganglion toxicity through various mechanisms,[11,12] including tubulin polymerization[13] and disturbances in calcium homeostasis.[14] The initial standard administration schedule for BTZ has been twice a week intravenously (IV) with at least 72 hours between doses, which results in a high incidence of new or worsening of pre-existing neuropathy. In both newly diagnosed and previously treated patients with multiple myeloma, reported incidences of neuropathy vary from 29% to 64%, and need for dose reduction for neurotoxicity varies from 12 to 19%.[7–10,15] In the VISTA trial, after a median of 8 cycles (46 weeks) of treatment with IV BTZ given twice weekly for newly diagnosed myeloma, 31% patients developed mild neuropathy (Grades1 and 2) and about 14% patients developed severe neuropathy (Grades 3 and 4).[7] Ten percent of enrolled patients had pre-existing neuropathy. In patients with relapsed and refractory myeloma, when BTZ was given twice weekly via IV route at the standard dose of 1.3 mg/m^2^, 23% patients developed mild treatment related neuropathy, while 14% developed severe neuropathy. With a lower dose of 1 mg/m^2^, 14% patients developed mild neuropathy and 7% were noted to have severe PN.[10] Eighty-three percent of these patients had pre-existing neuropathy and prevalence of treatment emergent neuropathy in this cohort increased with cumulative dosing and reached a plateau at cycle 5.[10]

When given intravenously, decreasing the frequency of administration to once a week causes less neuropathy without loss of efficacy. Only 27% of patients with newly diagnosed multiple myeloma randomized to once-weekly IV BTZ developed neuropathy, which was severe (grade 3 or 4) in 4%, compared to 46% PN (21% grade 3 and 4) with twice-weekly IV BTZ.[16] The median treatment duration prior to onset of neuropathy in the once weekly group was 4.4 months versus 3.5 months in the twice weekly group. In patients with light chain amyloidosis, weekly administration resulted in neuropathy in 22% of patients compared to 35% incidence in the twice weekly group in a randomized study.[5] BTZ administered twice-weekly via subcutaneous (SC) route is also non-inferior in efficacy to IV BTZ and results in less PN of all grades, 38% vs. 53%, respectively, and 6% vs. 16% for severe PN in a randomized controlled study in myeloma.[17] Additionally, SC BTZ is associated with better rate of resolution of neuropathy after treatment discontinuation, though time to resolution is similar.[18]

In an attempt to further reduce neuropathy, BTZ has been administered weekly via SC route at our institution since December 2010, for both upfront and subsequent treatment of multiple myeloma and AL amyloidosis. The objective of this retrospective study was to determine the incidence of neuropathy and efficacy of weekly SC BTZ compared to other schedules in patients closely followed at our institution.

## Methods

Following approval by the Cleveland Clinic Institutional Review Board including waiver of consent, patients with multiple myeloma and AL amyloidosis were identified from our plasma cell disorder database. Research reported in this study was conducted in accordance with the Declaration of Helsinki.

### Inclusion and exclusion criteria

Patients older than 18 years with multiple myeloma, AL amyloidosis or both who received BTZ for the first time, either as initial treatment or later during their disease course, and who started treatment between January 1^st^ 2005 and March 31^st^ 2013 were included. BTZ treatment alone or in combination with glucocorticoids or other chemotherapeutic agents was allowed. Patients who were not followed at our center (Cleveland Clinic in Cleveland, Ohio) were excluded.

### Study variables

The Electronic Medical Record (EMR) was analyzed for each patient and information was stored in RedCap software.[19] Data pertaining to the following variables were collected—demographics, diagnosis, underlying diseases known to predispose to neuropathy (specifically diabetes mellitus, hypothyroidism, amyloidosis, spinal stenosis, spinal degenerative disease, localized nerve impingement syndromes including carpal tunnel syndrome). For each BTZ based regimen the following data were collected- treatment dates, dose, route and frequency of BTZ administration, other concurrently administered drugs, known neurotoxicity of co-administered drugs, route of administration of BTZ, number of prior regimens, presence of neuropathy prior to treatment, grade of pre-existing neuropathy, development of new or worsening of pre-existing PN on treatment and its severity, best response to treatment and reason for treatment discontinuation. The course of neuropathy after treatment discontinuation was also noted.

### Definitions

Grade 1 and 2 neuropathy based on common terminology of adverse events criteria, National Cancer Institute (CTAE NCI) was regarded as mild neuropathy and grade 3 and 4 neuropathy was considered severe.[20] Severity of neuropathy was documented according to physician assigned grades noted in the EMR. If such grades were not clear from physician documentation, retrospective assessment was made based on description of functional impact of neuropathy, presence and intensity of pain, need for medications to treat neuropathy, and if treatment was discontinued as a consequence of neuropathy. Neuropathy with impairment of activities of daily living (ADL), severe pain and neuropathy which directly resulted in treatment discontinuation was considered severe. Impact of neuropathy on treatment, including dose reduction, decreased frequency of administration, treatment discontinuation and administration of drugs to treat neuropathy and their effectiveness in symptom control was recorded. Data was abstracted by 2 users and inter-rater verification was done for random samples.

One of our goals was to evaluate how long bortezomib could be tolerated without dose or schedule adjustment and whether the starting administration schedule influenced how long it could be given overall. For the purpose of this analysis two types of treatment durations were defined: treatment “segments” were defined primarily as a specific BTZ schedule based on frequency, dose and route of administration; treatment “intervals” were defined as a period of continuous BTZ treatment defined as treatment segments that were chronologically separated by ≤30 days. As an example, if a patient received BTZ weekly IV for 2 months, had treatment held for 10 days and then restarted treatment on a weekly SC schedule or if another drug was added, the patient would have two treatment segments, but one treatment interval. All BTZ based treatments after the initial regimen were analyzed to assess the treatment intervals.

Response assessment was according to International Myeloma Working Group (IMWG) consensus criteria for multiple myeloma and standard hematologic response criteria for amyloidosis. [21,22]

### Statistical analysis

Categorical variables were summarized as frequency counts and percentages; measured values as medians and ranges. Time to progression was measured from the start of treatment to the date of progression or last contact. Univariable analysis was carried out using chi-square tests for categorical data and the Kruskal-Wallis test for measured factors and factors with a natural ordering (e.g. response). Multivariable logistic regression models were used to assess the impact of treatment schedule (weekly SC BTZ versus other schedules) on neuropathy and response to treatment while adjusting for age, underlying disease associated with neuropathy, diagnosis, co-administration of other neurotoxic drugs and number of prior treatments received. All analyses were conducted using SAS version 9.4 (SAS Institute, Cary, NC).

## Results

**Fig 1:**
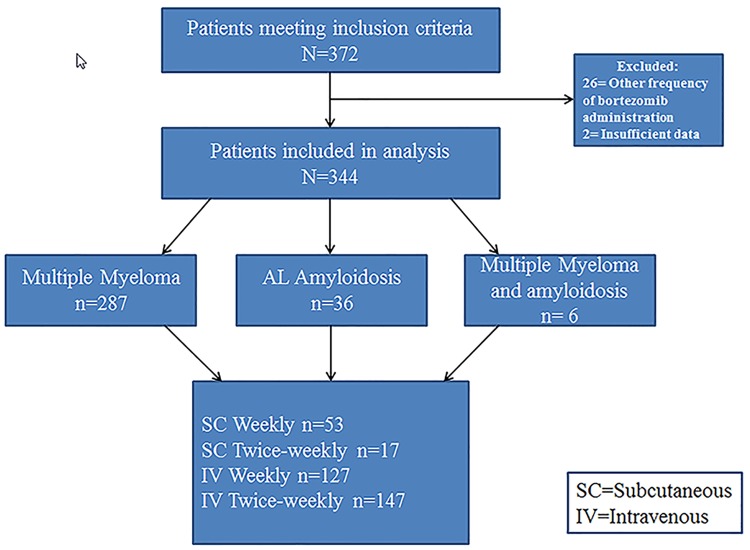
Consort flow diagram. Shown are patients pulled from our plasma cell disorder registry that fulfilled search criteria for first bortezomib containing regimen for myeloma or AL amyloidosis started between 1/1/2005 and 3/30/2013 and distribution of patients between analysed administration schedules.

We identified 372 patients meeting the search criteria for first bortezomib-containing regimen. Of these, 26 were excluded from analysis as the frequency of BTZ administration was other than once or twice weekly and two were excluded due to insufficient data (Fig 1). The remaining 344 patients were included in this analysis and began their first BTZ regimen between February 2005 and March 2013.

### Baseline characteristics and treatment duration

Baseline characteristics are listed in Table 1. Fifty-four percent (187/344) of patients were female and median age at the start of the first BTZ regimen was 62 years (range 30–89). Most patients (83%, 285/344) had myeloma, 10% (38/344) had amyloidosis, and 6% (21/344) were diagnosed with both. The majority of patients (61%, 210/344) received BTZ as part of their initial myeloma therapy. Overall, 58% (199/344) of patients had multiple BTZ treatment segments, meaning the dose, route or schedule were changed, and 30% (104/344) had multiple BTZ treatment intervals (regimens > 30 days apart), of which only the first interval is described here. The median duration of the first segment was 3.7 months (range 0.1–28.8); median duration of first treatment interval was 4.9 months (range 0.1–49.5).

**Table 1:** Baseline characteristics.

	All patients	SC Weekly	SC Twice Weekly	IV Weekly	IV Twice Weekly	P-value*
	N = 344	N = 53	N = 17	N = 127	N = 147	
Gender, Female: male	187:157	28:25	7:10	69:58	53:94	0.29
	(54%)	(53%)	(41%)	(54%)	(36%)	
Median age at start of bortezomib in years (range)	62	67	66	62	60	0.005
	(30–89)	(41–87)	(40–77)	(30–89)	(36–85)	
**Diagnosis**						
Myeloma	285	37	17	90	143	0.01
	(83%)	(70%)	(100%)	(70%)	(97%)	
Amyloidosis	36	11	0	23	2	
	(10%)	(21%)		(19%)	(2%)	
Myeloma + Amyloidosis	21	5	0	14	2	
	(6%)	(9%)		(11%)	(1%)	
**Conditions which pre-dispose to neuropathy, N (%)** *						
	156	28	2	74	53	0.29
	(45%)	(53%)	(12%)	(58%)	(36%)	
Diabetes	44	8	2	13	21	
	(13%)	(16%)	(20%)	(10%)	(14%)	
Amyloidosis	57	16	-	37	4	
	(17%)	(30%)		(30%)	(3%)	
Hypothyroidism	36	5		17	14	
	(11%)	(9%)		(13%)	(10%)	
Spinal disorders	25	3		12	10	
	(7%)	(6%)		(10%)	(7%)	
Carpal Tunnel Syndrome	1	-	-	5	5	
	(3%)			(4%)	(3%)	
Others	7	1	1	3	2	
	(2%)	(2%)	(6%)	(2%)	(1%)	
**Co-administration of neurotoxic drugs other than lenalidomide**						
Thalidomide	8	-	1	-	7	
	(2%)		(6%)		(5%)	
Cisplatin	1	-	-	-	1	
	(0.3%)				(1%)	
**Pre-existing neuropathy**						
Any severity	80	16	1	31	32	0.22
	(23%)	(30%)	(6%)	(24%)	(22%)	
Causes of pre-existing neuropathy**						
Diabetes	7	3	1	2	2	
	(2%)	(6%)	(6%)	(1%)	(1%)	
Thalidomide	31	3	-	9	19	
	(9%)	(6%)		(7%)	(13%)	
Amyloidosis	12	4	-	7	1	
	(4%)	(8%)		(6%)	(1%)	
MGUS	4	3	-	1	-	
	(1%)	(6%)		(1%)		
Others/Not known	35	8	-	17	10	
	(10%)	(15%)		(13%)	(7%)	
**Number of prior chemotherapy regimens**						
None	210	37	16	88	69	0.19
	(61%)	(70%)	(94%)	(69%)	(47%)	
One prior regimen	64	7	1	17	39	
	(19%)	(13%)	(6%)	(13%)	(27%)	
More than one prior regimen	70	9	-	22	39	
	(20%)	(17%)		(17%)	(27%)	

Abbreviations: SC = subcutaneous, IV = intravenous;

*SC weekly vs. other regimens.

**Some patients had more than one condition which pre-disposes to neuropathy/ caused previous neuropathy.

In the first treatment segment, 15% (53/344) of patients received BTZ SC weekly, 5% (17/344) SC twice weekly, 37% (127/344) IV weekly and 43% (147/344) IV twice weekly. In the majority of patients (95%, 305/321) the standard 1.3 mg/m^2^ dose was given independent of schedule. BTZ was used with glucocorticoids (dexamethasone or prednisone) in more than half the patients (55%, 190/344) and in 23% of patients (79/344) lenalidomide was also added.

As described in Table 1, patients receiving SC weekly BTZ were older with a median age of 67 years compared to 66 years for SC twice weekly, 62 years for IV weekly and 60 years for IV twice weekly regimens (p = 0.005). As listed in Table 1, there was no statistically significant difference in the proportion of patients with pre-existing neuropathy amongst the different groups. Thirty percent (n = 16) of patients in the SC weekly group had underlying neuropathy compared to 24% (n = 31) in the IV weekly group and 22% (n = 32) in the IV twice weekly group. There was only one patient in the SC twice weekly group who had pre-existing neuropathy. (p = 0.22). Causes of underlying neuropathy (n = 80) included prior thalidomide (n = 31, 39%), amyloidosis (n = 12, 15%), spinal disease (n = 11, 14%), diabetes (n = 7, 9%), monoclonal gammopathy related (n = 4, 5%) and others or unknown in the remainder (n = 24, 31%). (Table 1)

SC weekly BTZ could be continued longer, with median duration of both treatment segment (p = 0.004) and treatment interval (p = 0.001) significantly longer than with IV or SC twice weekly administration (Table 2). Patients were on SC weekly BTZ for a median of 4.7 months (range 0.2–28.8) in the first treatment segment and bortezomib could be administered without interruption (by ≥ 30 days) for 6.7 months (range 0.2–41.5) when treatment was initiated in a weekly SC schedule.

**Table 2:** Neuropathy and treatment response with SC weekly and other treatment schedules.

	Overall	SC Weekly	SC Twice Weekly	IV Weekly	IV Twice Weekly	P value*
**First bortezomib regimen**						
Bortezomib +/- steroids	214	34	10	81	89	0.32
	(62%)	(64%)	(59%)	(64%)	(60%)	
Bortezomib + lenalidomide +/-steroids	79	13	6	26	34	
	(23%)	(25%)	(39%)	(20%)	(23%)	
Bortezomib + other	51	6	1	20	24	
	(15%)	(11%)	(6%)	(16%)	(16%)	
**Duration of treatment**						
First treatment segment months, median (range)	3.7	4.7	3	4.4	3	0.004
	(0.1–28.8)	(0.2–28.8)	(0.4–9)	(0.1–22.1)	(0.1–19.9)	
First treatment interval months, median (range)	4.9	6.7	5	5.8	3.9	0.001
	(0.1–49.5)	(0.2–41.5)	(1.9–27.6)	(0.1–42.4)	(0.1–49.5)	
**Neuropathy with bortezomib** **						
No neuropathy	143	27	11	59	46	0.001
	(42%)	(52%)	(65%)	(47%)	(32%)	
Pre-existing, stable	44	11	1	18	14	
	(13%)	(21%)	(6%)	(14%)	(10%)	
Worsening of pre-existing neuropathy	34	5	0	12	17	
	(10%)	(10%)		(10%)	(12%)	
New neuropathy	119	9	5	36	69	
	(35%)	(17%)	(29%)	(29%)	(47%)	
**Impact of neuropathy**						
Treatment stopped	63	4	1	18	39	0.11
	(32%)	(16%)	(17%)	(27%)	(39%)	
Drugs to treat neuropathy	64	8	3	12	40	1.0
	(32%)	(32%)	(50%)	(18%)	(40%)	
Dose reduction	31	2	1	10	18	0.38
	(16%)	(8%)	(17%)	(15%)	(18%)	
Decreased frequency of administration	21	0	1	0	20	0.08
	(11%)		(17%)		(20%)	
Improvement/Resolution of neuropathy after treatment discontinued (N = 133)***						
No improvement	17	2	0	7	8	0.71
	(13%)	(18%)		(17%)	(10%)	
Partial Improvement	77	6	2	19	50	
	(58%)	(55%)	(50%)	(46%)	(65%)	
Complete resolution	39	3	2	15	19	
	(29%)	(27%)	(50%)	(37%)	(25%)	
**Response to treatment in 287 evaluable patients**						
CR	32	6	2	10	14	0.15
	(11%)	(13%)	(14%)	(9%)	(12%)	
VGPR	94	20	5	37	32	
	(33%)	(43%)	(36%)	(33%)	(28%)	
PR	83	10	3	36	34	
	(29%)	(21%)	(21%)	(32%)	(30%)	
SD/MR	59	10	4	23	22	
	(21%)	(21%)	(29%)	(21%)	(19%)	
PD	19	1	0	5	13	
	(7%)	(2%)		(5%)	(11%)	
ORR	209	36	10	83	80	
	(72.8%)	(76.6%)	(71.4%)	(74.8%)	(69.6%)	

Abbreviations: CR = complete remission, VGPR = very god partial remission, PR, partial remission, SD = stable disease, MR = minor response, PD = progressive disease, ORR = overall response rate according to IMWG uniform response criteria ^21^.

*P value: SC weekly vs. other regimens,

**Data not available for 4 pts,

***Data not available for 12 patients.

### Risk of neuropathy

To avoid selection bias, only the first BTZ segments were compared for toxicity and response in this analysis (Table 2). There was no difference in pre-existing neuropathy and co-morbidities associated with neuropathy among the four treatment groups; 45% (156/344) of all patients had comorbidities associated with neuropathy at the start of the initial BTZ treatment, primarily amyloidosis (n = 59), diabetes (n = 44), and/or hypothyroidism (n = 32). Neuropathy was already present in 23% of patients (80/344), and was generally mild (94%, 75/80). There was also no difference in proportion of patients who received other neurotoxic drugs along with BTZ (Table 1), which included lenalidomide in the majority (90%), followed by thalidomide (9%) and cisplatin (1%). When all treatment regimens were analyzed together, pre-existing neuropathy worsened (any grade) in 43% of patients (34/80) with BTZ treatment and 45% (119/264) of patients without prior neuropathy developed it. Development of symptoms suggestive of neuropathy and worsening of prior symptoms were significantly lower in the SC weekly group compared to the other groups (p = 0.001 for new neuropathy, p = 0.006 for both new and worsening of prior neuropathy). Symptoms of neuropathy with bortezomib were seen in 27% patients in the SC weekly group (new neuropathy = 17%; worsening of prior neuropathy = 10%), compared to 29% in the SC twice weekly group and 39% and 59% in the IV weekly and IV twice weekly cohorts, respectively. (Table 2)

Table 3 describes multivariable analysis for development of neuropathy. Even when adjusted for other factors in the multivariable analysis, treatment regimens other than SC weekly, were twice as likely to result in neuropathy or worsening of pre-existing neuropathy (2.45, 95% Confidence Interval 1.26–4.76, p = .008). There was also some indication that co-administration of other neurotoxic drugs (thalidomide, vincristine, etc.) increased the risk of neuropathy (odds ratio 1.64, 95% confidence interval 1.00–2.68; p = .05). There was no indication however of increased risk due to age, number of prior chemotherapy regimens or the presence of underlying diseases associated with neuropathy.

**Table 3:** Multivariable analysis for development of new neuropathy and worsening of prior neuropathy.

Variable	Odds Ratio (95% CI)	P value^1^
Bortezomib schedule		0.008
SC weekly	Reference	
Others	2.45 (1.26–4.76)	
Age in decades^2^	0.92 (0.75–1.12)	0.41
Underlying disease which may cause neuropathy		0.97
No	Reference	
Yes	1.01 (0.65–1.57)	
Co-administration of neurotoxic agents		0.05
No	Reference	
Yes	1.64 (1.00–2.68)	
Prior treatments (0 vs. 1 vs. >1)^2^	0.96 (0.73–1.27)	0.79

^1^ Wald test

^2^ The odds ratio represents the impact on neuropathy for a one unit increase in the variable

As shown in Table 2, 77% of patients showed an improvement in neuropathy after treatment was discontinued, with complete resolution of symptoms in 29%. There was no difference seen across different treatment schedules. Development of neuropathy often led to administration of drugs like duloxetine, gabapentin or amitriptyline to treat neuropathic pain. Thirty-two percent of patients in the SC weekly group (n = 8) required medications compared to 50% (n = 3) in SC twice weekly, 18% (n = 12) in IV weekly, and 40% (n = 40) in IV twice weekly groups, but there was no statistically significant difference between SC weekly and other groups. PN resulted in BTZ dose reduction in 8% of SC weekly (n = 2), 17% of SC twice weekly (n = 1), 15% of IV weekly (n = 10) and 18% of IV twice weekly treated patients (n = 18). Decreased frequency of administration was mainly observed in the twice weekly IV group (20%, n = 20). Treatment was discontinued due to neuropathy in 16% patients in the SC weekly group (n = 4), in 17% of patients in the SC twice weekly group (n = 1), in 27% of subjects receiving once weekly IV BTZ (n = 18) and in 39% receiving it IV twice weekly IV (n = 39).

### Response evaluation

Overall, 11% (32/287) of patients achieved a bone marrow biopsy confirmed complete response (CR), 33% (94/287) had a very good partial response (VGPR) including immunofixation negative state without bone marrow exam, 29% (83/287) achieved a partial response (PR), 21% (59/287) had a best response of stable disease (SD) or minor response (MR) or No Response (for patients with amyloidosis only), and 7% (19/287) of patients progressed on treatment (Table 2). Response was similar (CR, VGPR or better and PR or better) across all four treatment schedules in univariable analysis and when other factors, including prior treatment, were accounted for in multivariable analysis (Table 4). SC weekly dosing resulted in an overall response rate (ORR) (partial response or better) of 77% including CR in 13% patients and VGPR in 43% patients. Although ORR did not differ by schedule, patients receiving bortezomib as initial therapy had a higher likelihood of achieving PR or better (p-0.0002) compared to patients who had received prior therapy for myeloma or amyloidosis. Younger patients also had a higher likelihood of achieving response (p = 0.07).

**Table 4:** Multivariable analysis for failure to achieve at least partial response.

Variable	Odds Ratio (95% C.I.)	P value^1^
Schedule		
SC weekly	Reference	0.56
Others	1.25 (0.58–2.71)	
Age in decades^2^	1.30 (1.01–1.68)	0.04
Prior treatment regimens (0 vs. 1 vs. >1)^2^	1.88 (1.36–2.58)	0.0001
**Diagnosis**		
Myeloma	Reference	
Myeloma+ Amyloidosis	1.43 (0.46–4.47)	0.54
Amyloidosis	1.03 (0.35–3.05)	0.95

^1^ Wald test

^2^ The odds ratio represents the impact on response for a one unit increase in the variable

Factors in the multivariable model included: Treatment schedule, diagnosis, age in decades, and number of prior chemotherapy regimens. Results were similar with models for Complete Response (CR) and VGPR or better response.

To assess whether progression-free survival might be affected by the bortezomib administration schedule we performed multivariable analyses that adjusted for underlying disease (myeloma vs. AL amyloidosis) and prior therapy (newly diagnosed vs. relapsed or refractory) and found no statistically significant difference (p = 0.17).

A small cohort of our patients (n = 26) received SC weekly BTZ with dexamethasone or prednisone only as upfront treatment for multiple myeloma or amyloidosis. Of these, 22 patients (18 with myeloma with or without amyloidosis and 4 with systemic amyloidosis alone) were evaluable for response and 18/22 (82%) achieved PR or better, including 13 (59%) with at least VGPR. Five of them (23%) developed new neuropathy, which was mild in all cases and none of the 7 patients with pre-existing neuropathy developed any worsening of symptoms.

Overall, our results demonstrate that SC weekly treatment with BTZ is tolerated longer with less neuropathy and results in equally good response compared to other administration schedules.

## Discussion

Multiple myeloma and amyloidosis predispose patients to neuropathy from multiple causes, including iatrogenic factors. Treatment with drugs such as thalidomide, previously vincristine and now bortezomib (BTZ), results in high incidence of neuropathy in this patient population.[23] Bortezomib is highly active in both multiple myeloma and amyloidosis, but PN often limits prolonged use. Although IV once weekly and SC twice weekly schedules are associated with less neurotoxicity compared to IV twice weekly administration, neuropathy still develops in a large proportion of patients resulting in dose reduction or discontinuation of treatment, potentially limiting therapeutic benefit. Many institutions have now adapted the once weekly subcutaneous schedule, though there is limited data comparing this schedule to others and the risk of neuropathy with this schedule.[24–26]

Our retrospective study suggests that there is a significantly lower incidence of neuropathy when bortezomib is used once weekly via the SC route compared to other schedules without compromising efficacy of treatment. Patients in all treatment groups had similar relevant baseline characteristics like pre-existing neuropathy or diseases known to predispose to neuropathy, as well as co-administration of neurotoxic drugs. Findings were consistent for both myeloma and amyloidosis. Overall, nearly half (45%) of our cohort of 344 patients developed symptoms suggestive of new neuropathy or worsening of pre-existing neuropathy (any grade). Comparable to previous reports [6,10,15] this was seen in 59% of patients treated IV twice a week and 39% of patients in the weekly IV group, whereas only 27% of SC weekly treated patients suffered new or worsening PN (p = 0.001). While our SC twice a week BTZ group was too small to draw conclusions, our data compare favorably to a reported PN incidence of 38% for twice weekly SC BTZ administration.[17] Although there was a trend of increased neuropathy in the presence of conditions known to cause neuropathy like diabetes, it did not reach statistical significance. This is consistent with prior reports which have shown that co-existing conditions and prior neurotoxic treatments do not impact risk of developing neuropathy.[10,16] Neither response to treatment (Table 2), 77% for SC weekly BTZ which is comparable to response rates using established dosing,[5,16] nor progression-free survival appeared negatively affected by the SC weekly administration schedule.

Rate of neuropathy in a phase II study with once weekly SC BTZ, cyclophosphamide and dexamethasone was 22% in a cohort of 31 relapsed-refractory myeloma patients, of which 52% had been previously exposed to bortezomib. This incidence is lower than our study. The slightly lower rate of neuropathy may be secondary to the fact that half the study subjects had been exposed to bortezomib before and it is possible that they had a tendency to tolerate bortezomib better.[26] A recent retrospective study challenges lower neurotoxicity with SC vs. IV administration of BTZ.[25] Importantly, the authors did not analyze weekly vs. twice a week separately and excluded patients in their analysis who switched from twice a week to weekly and between administration routes. The analyzed patient population predominantly received weekly BTZ (63%) and achieved both comparable disease control and comparable neurotoxicity independent of administration route.[25] To avoid selection bias we did account for intra-patient changes in treatment route and frequency in our analysis, but on direct comparison of SC vs. IV weekly we also did not find statistically significant differences in neuropathy, although a higher rate of treatment discontinuation with IV administration (27% vs. 16%, Table 2) suggests that a larger study might have identified a statistically significant change. It has previously been shown in randomized trials that SC BTZ when given twice weekly carries lower neurotoxicity risk than when given IV (Moreau et al 2011).[17] It has also been shown that once weekly dosing of IV BTZ causes less neuropathy than twice weekly dosing.[16] Our findings are consistent with results of these trials and were obtained from electronic medical records of patients treated by a team of physicians who routinely grade neuropathy for clinical studies and document key aspects required for grading like functional consequences of PN or use and adjustment of drug regimens used to treat painful PN.

Long-term tolerability of BTZ is essential, given evidence that consolidation and maintenance treatment with BTZ in myeloma is associated with improved progression-free survival, [27,28] and possibly improved overall survival as well.[27] When myeloma patients were surveyed on their opinion on maintenance treatment, development of peripheral neuropathy was the most common worrisome potential toxicity, identified by 27% of patients.[29] Our study suggests that use of SC weekly induction may overcome this barrier for many patients and allow maintenance at reduced frequency with likely further reduced neuropathy risk. SC administration also eliminates the need for IV access, reduces infusion chair times, cost and is preferred by patients.[30] The additional adverse effects of SC over IV administration are limited to a very low incidence of severe local site reactions.[17]

Our data from 22 response evaluable patients who received weekly SC BTZ with steroids only as upfront treatment document that this treatment, too, causes little neuropathy (23%) and can be very effective with 82% achieving PR or better and 59% VGPR or better. This combination is especially important for elderly and frail patients, where addition of a third drug may increase treatment toxicity.

At present, there are three proteasome inhibitors approved for the treatment of multiple myeloma with different toxicity profiles and preferred routes of administration. Carfilzomib and ixazomib have the advantage of causing less peripheral neuropathy. However, carfilzomib requires IV administration and the standard schedule of administration on days 1, 2, 8, 9, 15, and 16 of a 28 day cycle is relatively inconvenient. Importantly, the lower neuropathy risk of carfilzomib also comes at a higher risk for cardiotoxicity than BTZ.[31] Ixazomib is an oral (PO), reversible proteasome inhibitor similar to BTZ.[32–34] Rate of neuropathy with ixazomib when given in combination with lenalidomide and dexamethasone was 27% compared to 22% in the lenalidomide and dexamethasone arm.[32] However, ixazomib is associated with high rates of gastrointestinal toxicity.[32–34] At present there is no prospective trial comparing ixazomib and bortezomib. Which of the two drugs, ixazomib PO or bortezomib SC, provides the best therapeutic index when given on a day 1,8,15 every 28 day schedule is a question worth asking in the context of a clinical trial.

In conclusion, our analysis supports use of the weekly SC BTZ administration schedule since it showed activity comparable to less convenient established schedules with favorable neurotoxicity profile.
